# Publisher Correction: Self-growing photonic composites with programmable colors and mechanical properties

**DOI:** 10.1038/s41467-023-37723-2

**Published:** 2023-04-05

**Authors:** Juan Xue, Xuewu Yin, Lulu Xue, Chenglin Zhang, Shihua Dong, Li Yang, Yuanlai Fang, Yong Li, Ling Li, Jiaxi Cui

**Affiliations:** 1 Institute of Fundamental and Frontier Sciences, University of Electronic Science and Technology of China, No. 5, Section 2, North Jianshe Road, Chengdu, Sichuan 610057 P. R. China; 2 Yangtze Delta Region Institute (Huzhou), University of Electronic Science and Technology of China, Huzhou, 313001 P. R. China; 3 Department of Bioengineering, University of Pennsylvania, Philadelphia, 19104 USA; 4 Department of Mechanical Engineering, Virginia Polytechnic Institute and State University, 635 Prices Fork Rd, Blacksburg, VA 24060 USA

**Keywords:** Materials for optics, Structural materials

Correction to: *Nature Communications* 10.1038/s41467-022-35555-0, published online 19 December 2022

The original version of this Article contained an error in the first sentence of the Author Contributions Section, both in the html and in the PDF version. The sentence incorrectly read:

“C.Z and J.X. conceived the concept.”

While the correct form should be:

“J. C. and J. X. conceived the concept.”

This has been corrected in both versions of the Article.

