# Supplementary material for: Self-growing photonic composites with programmable colors and mechanical properties
Source: Nat Commun. 2022 Dec 19;13:7823. doi: 10.1038/s41467-022-35555-0 (PMC9763393; doi:10.1038/s41467-022-35555-0)
Supplement: Supplementary file 1 — Supplementary Information [file 41467_2022_35555_MOESM1_ESM.pdf]

Supplementary Information

Self-growing Photonic Composites with Programmable Colors and Mechanical Properties

Juan Xue[1, 2], Xuewu Yin[1], Lulu Xue[3] Chenglin Zhang[1], Shihua Dong[1], Li Yang[1], Yuanlai Fang[1], Yong Li[1], Ling Li[4], and Jiaxi Cui* [1, 2]

[1]Institute of Fundamental and Frontier Sciences, University of Electronic Science and Technology of China, No. 5, Section 2, North Jianshe Road, Chengdu, Sichuan, 610057, P. R. China;

[2]Yangtze Delta Region Institute (Huzhou), University of Electronic Science and Technology of China, Huzhou 313001, P. R. China;

[3]Department of Bioengineering, University of Pennsylvania, Philadelphia, Pennsylvania, 19104, United States

[4]Department of Mechanical Engineering, Virginia Polytechnic Institute and State University. 635 Prices Fork Rd, Blacksburg, VA 24060, USA.

*Corresponding author: Jiaxi.Cui@uestc.edu.cn

1. Supplementary Methods

Scanning electron microscope (SEM)

The samples were directly sputtered with gold before carrying out imaging. For transverse cross-section structure, samples were firstly prepared by freezing embrittlement of liquid nitrogen at the target positions. Furthermore, sputtered with gold before carrying out imaging. A field emission scanning electron microscope (FEI, INSPECT F) at an accelerating voltage of 20 kV was used.

Attenuated total reflection-Fourier transform infrared (ATR-FTIR)

ATR-FTIR measurements were recorded with a Bruker VERTEX 70v FTIR spectrometer. The samples were directly fixed on the ATR fixture. The test wavenumbers range was from 4000 to 600 cm^{-1}.

UV-Visible light spectra (UV-vis)

UV-vis reflectance spectra were obtained from a Varian Cary 4000 UV-visible spectrophotometer. The tested sample was placed directly on the solid fixture with the illuminated side facing the light inlet. The test wavelength range was from 200 to 900 nm.

Surface profile

The surface profiles of the specimen were carried out on a Contour GT-K1.

Infrared thermal imaging

Infrared thermal imaging was obtained with infrared thermal imager LS13D2-1156 and the incident angle was fixed at 0°.

Digital images

Optical microscope images were acquired from an iPhone Xs or a Nikon ECLIPSE LV100ND.

Tensile testing

The measurements were conducted on a universal testing machine (ZWICK 1446) with a load cell of 10 KN and crosshead velocity of 10 $mm\cdot min^{-1}$. The moduli were calculated in the linear elastic region of the stress-strain curves from 1 to 5%. Five specimens were tested for each condition.

Water contact angles testing

Static water contact angles of materials were collected using a OCA 35 goniometer (DataPhysics Instruments). The volume of droplets was ~3 μL.

2. The microstructure of a peacock's tail feather

The peacock's tail feathers were purchased online. We checked the structure of the feather by SEM.

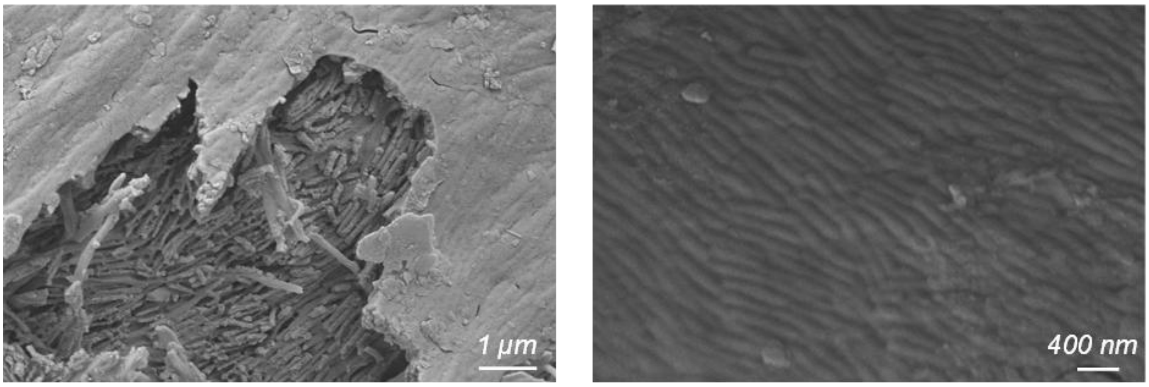

Supplementary Figure 1. Low (left) and high (right) magnification SEM images of the longitudinal cross-section of the peacock's brown barbule.

All colored barbules of peacock's tail leather show similar photonic crystals structures (**Figure 1A & Supplementary Figure 1**). They show various colors due to the differences in the lattice constants (rod spacing) along the direction normal to the cortex surface. The lattice constants for the blue are 140 nm and 150 nm for the brown barbule, which are consistent with the reported values (#29 literature in main text).

3. The chemical equations of the transesterification reaction

Chemical reaction equations

1. Polymerization:

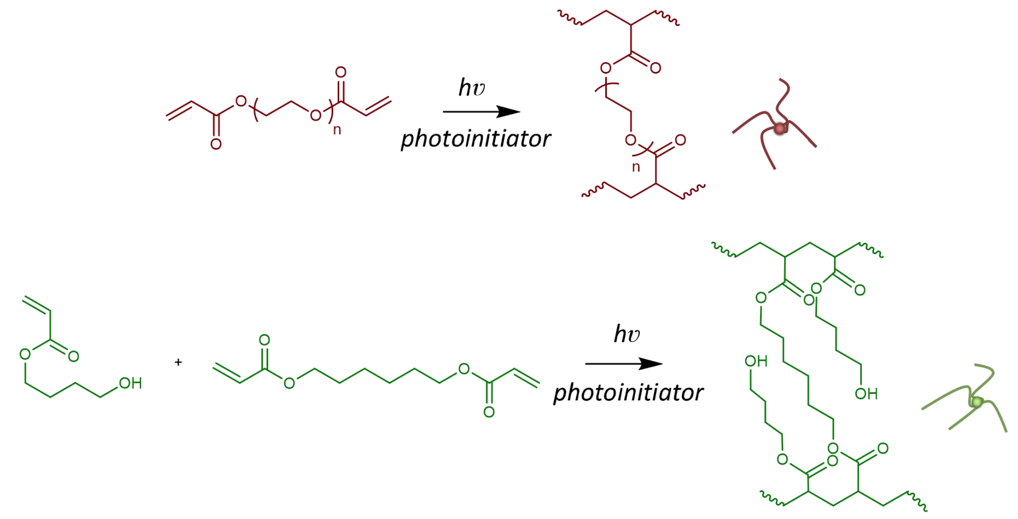

2. Transesterification:

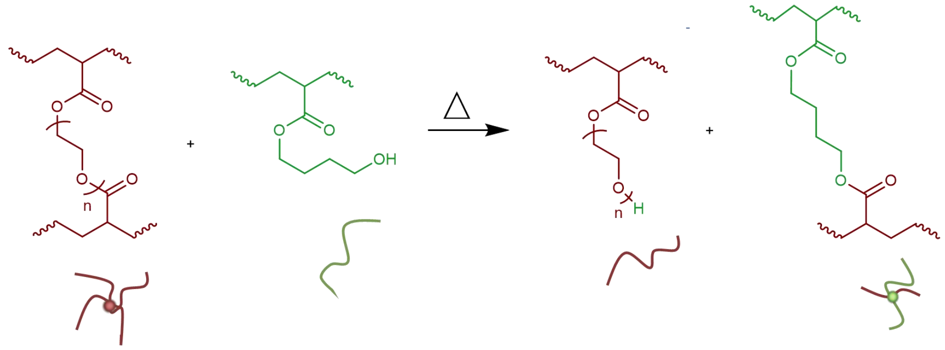

Supplementary Figure 2. 1. Initial (red one) and newborn (green one) polymer networks formed via photopolymerization. 2. The grown matrices are homogenized via transesterification reactions between the original and newborn polymer networks.

Here, we illustrate the chemical processes of initial network release using the initial sample EG_{40} grown in nutrient solution B as an example.

4. SiO_2 nanoparticles

Synthesis of SiO_2 nanoparticles

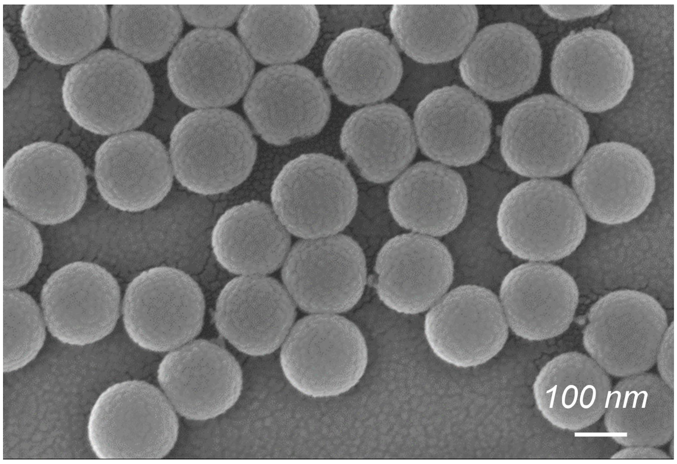

Supplementary Figure 3. SEM images of SiO_2 nanoparticles synthesized in our work. Spherical particles were observed.

The experimental data analysis in this research were performed with the statistical analysis software Nanomeasure. The diameters of at least ten independent SiO_2 nanoparticles were collected in **Supplementary Figure 3**. According to the statistical analysis from the SEM image, the average diameter of SiO_2 nanoparticles is ~200 nm.

The refractive index n (20°C) of SiO_2 nanoparticles

The refractive index *n* (20°C) of SiO_2 nanoparticles was acquired by the normalization of the individual *n* (20°C) values of its suspending solutions.

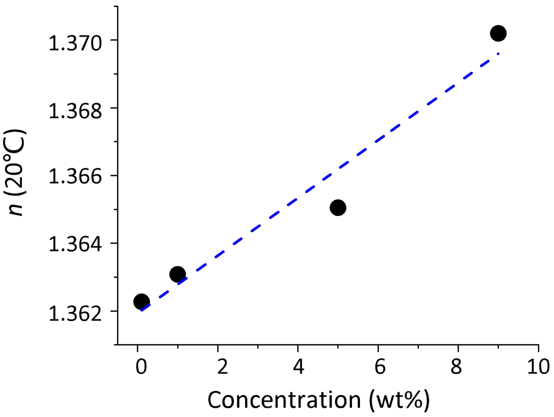

Supplementary Figure 4. The relationship curves of *n* (20°C) and concentrations of SiO_2 ethanol suspensions.

Ethanol was used as the dispersant to prepare a series of SiO_2 suspensions (SiO_2 concentrations: 0.1, 1, 5, and 9 wt%, respectively) for measurements. Their *n* (20°C) values were 1.36227, 1.36308, 1.36505, and 1.37020 (tested at the wavelength of 589 nm using Abbemat 300 refractometer), respectively. Through normalization (**Supplementary Figure 4**),

the relationship between n (20°C) and the concentrations of SiO_2 ethanol suspensions could be expressed as equation **Supplementary Equation 1**:

$$y = 0.0008517x + 1.3619 \qquad (\mathbf{1}),$$

When the concentration was 100 wt%, n (20°C) of synthesized SiO_2 was 1.45. In our system, the polymer showed a similar refractive index n with that of SiO_2 (polymer: 1.47 at 20°C), and therefore, the n contrast is reduced in grown films. Note that the optical transparency and reflectance exhibit a trade-off relationship (When the n contrast is reduced in photonic composite films, optical transparency can be improved, #39 literature in main text). Here, the thickness of the grown specimens is sufficient to obtain a saturated reflectance color (the reflectance intensities of the obtained growth sample films are above 45%) that can be observed by the human eye.

5. Fabrication of photonic composite films

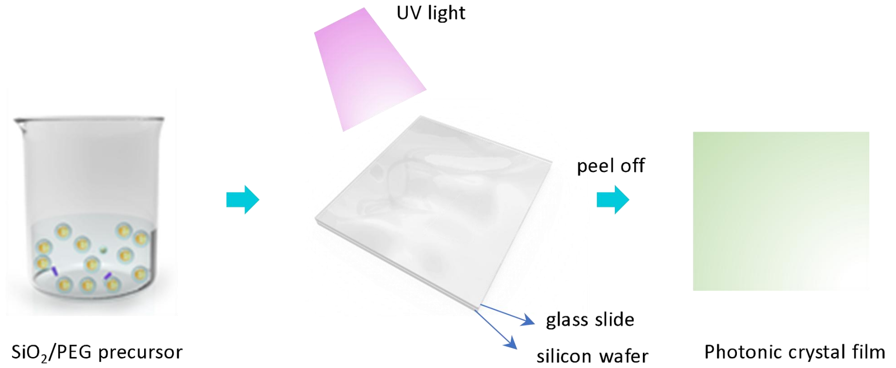

Supplementary Figure 5. A schematic image to describe the procedure of preparing a photonic composite film.

6. Swelling

The nutrient solution could be provided to the sample by two approaches: 1) immersing the sample in nutrient solution for allowing the sample to be fully swollen (12 h at room temperature); 2) drop the preset amount of nutrient solution on the sample and the solution would be absorbed into the film (this approach allowed for precisely controlling the swelling ratio). The weights of the obtained samples were collected to plot the swelling kinetics. Here the swelling ratio was calculated in **Supplementary Equation 2**:

$$Swelling\ ratio = \frac{W_{\text{swollen}} - W_{\text{dry}}}{W_{\text{dry}}} \qquad (\mathbf{2}),$$

where W_{swollen} is the weight of the swollen sample and W_{dry} is the weight of the dry sample.

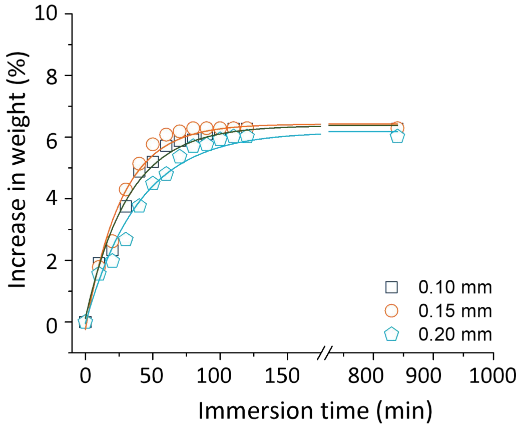

Supplementary Figure 6. Swelling curves of EG_{40} with average thicknesses of 0.10, 0.15 and 0.20 mm in EG.

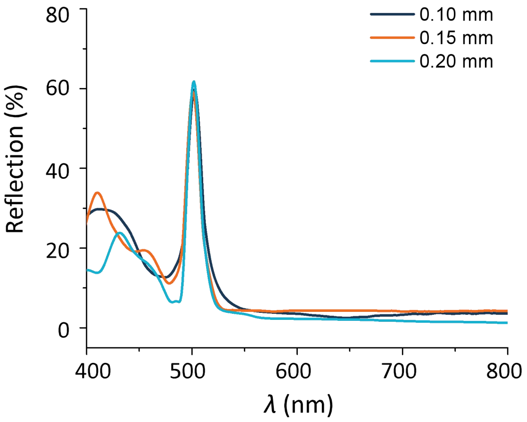

Supplementary Figure 7. UV-Visible spectra of grown $EG_{40} - EG_{10.6}$ obtained from EG_{40} with different thicknesses (0.10, 0.15, and 0.20 mm).

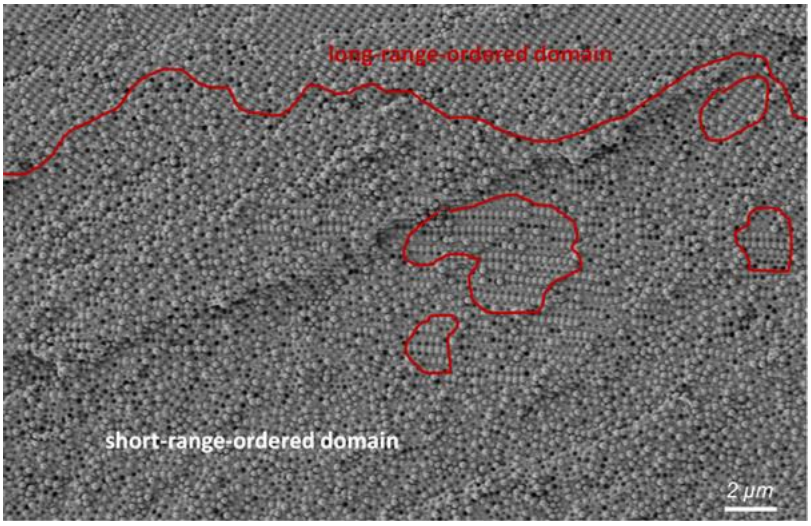

Supplementary Figure 8. SEM images of grown sample $EG_{40} - EG_{10.6}$ synthesized in our

work.

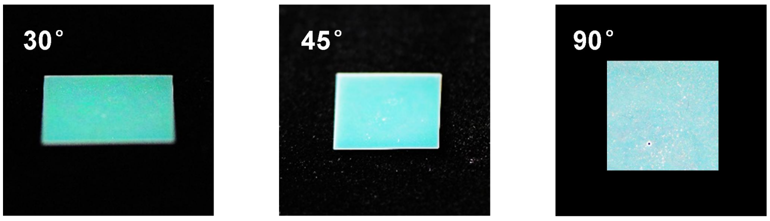

Supplementary Figure 9. Digital photos of $EG_{47.8}$ taken under different shooting angles (30°, 45°, and 90°).

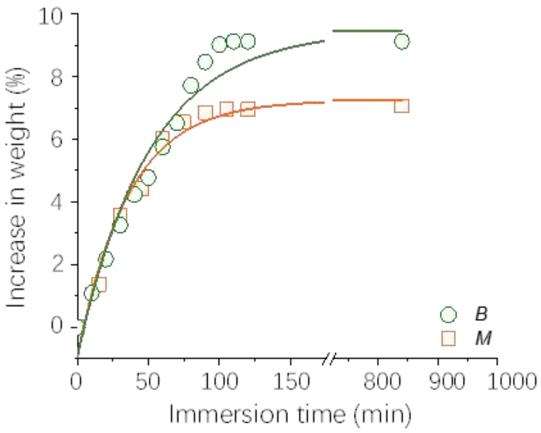

Supplementary Figure 10. Swelling curves of EG_{40} immersed in B and M.

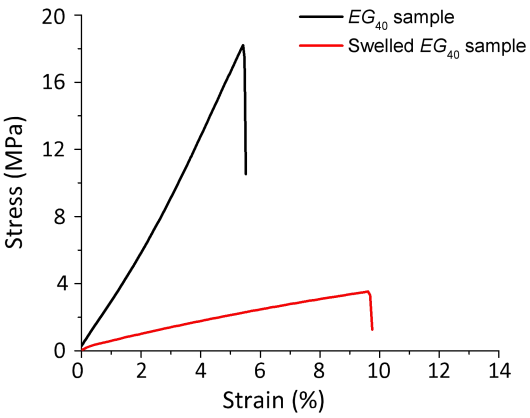

Supplementary Figure 11. Strain-stress curves of initial and swelled EG_{40} samples.

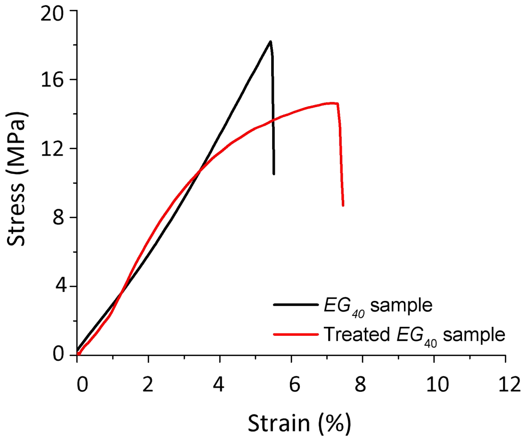

Supplementary Figure 12. Strain-stress curves of initial EG_{40} and EG_{40} swelled and subsequently irradiated by UV light (365 nm, 10 mW·cm^{-2}).

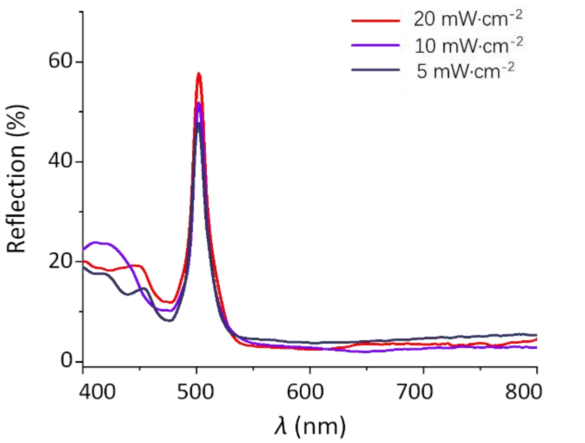

Supplementary Figure 13. UV-Visible spectra of the samples $EG_{40} - EG_{10.6}$ obtained from EG_{40} under different UV light irradiation intensities (5, 10, and 20 mW • cm^{-2}).

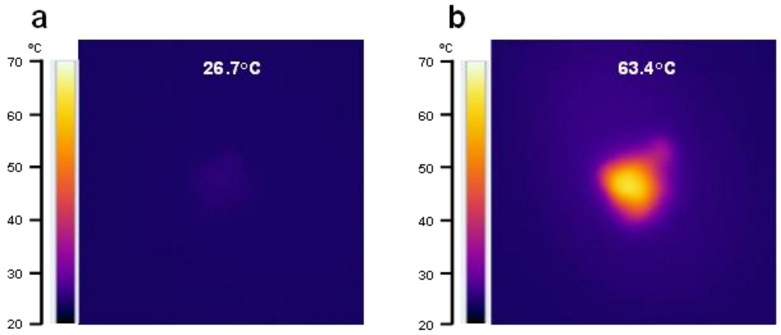

Supplementary Figure 14. Temperature changes of the swelled sample before (a) and after (b) UV irradiation for 50 s.

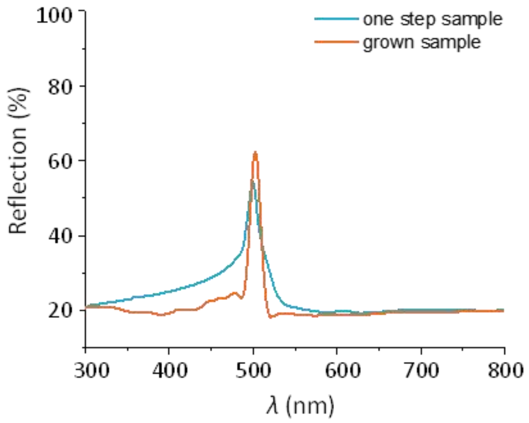

Supplementary Figure 15. UV-Visible spectra of the samples EG_{46} and $EG_{40}-EG_{10.6}$. EG_{46} was obtained by one-step polymerization. The EG_{46} has the same compositions with $EG_{40}-EG_{10.6}$ [(40+10.6)/(100+10.6)=45.8%]

7. Theoretically calculated interplanar spacing (2*d*) and reflection wavelengths (λ)

According to previous report (#38 literature in main text), the reflection wavelength (λ) of photonic composites with stacked hexagonal arrays corresponded to (111) plane was determined as follows:

$$m\lambda = 2d_{111}n_{\text{eff}} = \left(\frac{\pi}{3\sqrt{2}(1-\emptyset)}\right)^{\frac{1}{3}}\left(\frac{8}{3}\right)^{\frac{1}{2}} D\left(n_s{}^2(1-\emptyset)+n_{\text{poly}}{}^2\emptyset\right)^{\frac{1}{2}} \qquad \textbf{(3)},$$

where n_{eff} indicates the mean refractive index of the composites, *D* represents the average diameter of SiO_2 nanoparticles, n_s and n_{poly} are the refractive indices of SiO_2 nanoparticles and the polymer matrix, respectively, $\emptyset$ is the volume fraction of the polymer matrix. Since the refractive indices of SiO_2 nanoparticles (1.45) and the polymer matrix (1.457) are nearly the same, n_{eff} was considered as a constant. The **Supplementary Equation 3** can be simplified as follows:

$$2d_{111} = \left(\frac{\pi}{3\sqrt{2}(1-\emptyset)}\right)^{\frac{1}{3}}\left(\frac{8}{3}\right)^{\frac{1}{2}} D \qquad \textbf{(4)},$$

where proportional relation between $2d_{111}$ and the cube root of the volume fraction of SiO_2 nanoparticles ($1/\sqrt[3]{1-\emptyset}$) can be manifested. In our system, due to the existence of random domains, the *fcc* structure of photonic composites is slightly deformed. Simple geometrical analysis shows that interplanar spacing 2*d* is related to the interplanar distances d_{111} comprising the film according to **Supplementary Equation 5**,

$$2d = D + \sqrt{3}d_{111} \qquad \textbf{(5)},$$

Therefore, we modified the equation combining **Supplementary Equation 4** with **Supplementary Equation 5** as follows:

$$2d = R\frac{1}{\sqrt[3]{1-\emptyset}} + A = R\frac{1}{\sqrt[3]{\frac{M_S\rho_{poly}}{M_S\rho_{poly}+M_{poly}\rho_S}}} + A \qquad (6),$$

where M_S and M_{poly} are the mass fraction of the SiO_2 nanoparticles and the polymer matrix, ρ_s and ρ_{poly} are the densities of the SiO_2 nanoparticles and the polymer matrix, *R* and *A* are constants for a given system (depends on the diameter of SiO_2 nanoparticle). By using the data obtained from the initial photonic composite EG_{40} (ρ_s: 2.2 g·cm^{-3}; ρ_{poly}: 1.1 g·cm^{-3}; D: 183 nm), *R* of 610 and *A* of -512 were obtained. The remaining value of grown samples were calculated by **Supplementary Equation 7**:

$$2d = 610\frac{1}{\sqrt[3]{1-\emptyset}} - 512 \qquad (7),$$

The values were used to get the theoretic curve in **Figure 2F** in the main text.

Also, the theoretical λ was calculated by using a n_{eff} of 1.453 and equation #1 in the main manuscript. The values were used to get the theoretic curve in **Figure 2G**.

The $1-\emptyset$, normalized $1/\sqrt[3]{1-\emptyset}$, theoretical 2*d*/λ, experimental 2*d*/λ were shown in **Supplementary Table 1**.

Supplementary Table 1. The calculation values of $1-\emptyset$, $1/\sqrt[3]{1-\emptyset}$, theoretical 2*d*/λ and experimental 2*d*/λ of EG_{40} and its grown products.

Sample	$1-\emptyset$	$(1-\emptyset)^{-\frac{1}{3}}$	Theoretical 2*d* (nm)	Experimental 2*d* (nm)	Theoretical λ (nm)	Experimental λ (nm)
EG_{40}	0.42	1.34	305.24	313 ± 7.5	454.79	481
$EG_{40}-EG_{10.6}$	0.36	1.41	347.95	342 ± 8.2	496.93	502
$EG_{40}-EG_{22.8}$	0.31	1.48	390.67	370 ± 10.3	537.61	535
$EG_{40}-EG_{29.7}$	0.29	1.51	408.97	402 ± 7.1	584.11	582
$EG_{40}-EG_{37.2}$	0.27	1.55	433.38	436 ± 10.5	633.51	633
$EG_{40}-EG_{45.4}$	0.25	1.59	457.79	473 ± 9.2	687.27	690

8. Ester-linkage between SiO_2 and polymer matrices

We assumed that ester-linkages would form between the hydroxyl group on the surface of SiO_2 nanoparticles and the polymer matrices (by transesterification reactions even in the absence of any catalyst), during the fabrication. A control experiment was designed to prove this hypothesis. The SiO_2 nanospheres (0.308 g) were dispersed in an ethanol solution (2 mL) containing ethyl decanoate (140 μL), and the mixture was stirred at 70°C for 4 h.

Subsequently, the products were collected by high-speed centrifugation (a rate of 9000 r·min^{-1} for 10 minutes) and washed with *n*-hexane 3 times. The resulting products were dried in an oven at 60°C. The intact SiO_2 nanoparticles and ethyl decanoate-treated SiO_2 nanoparticles were both pressed into sheets with a diameter of 1 mm for FTIR measurements.

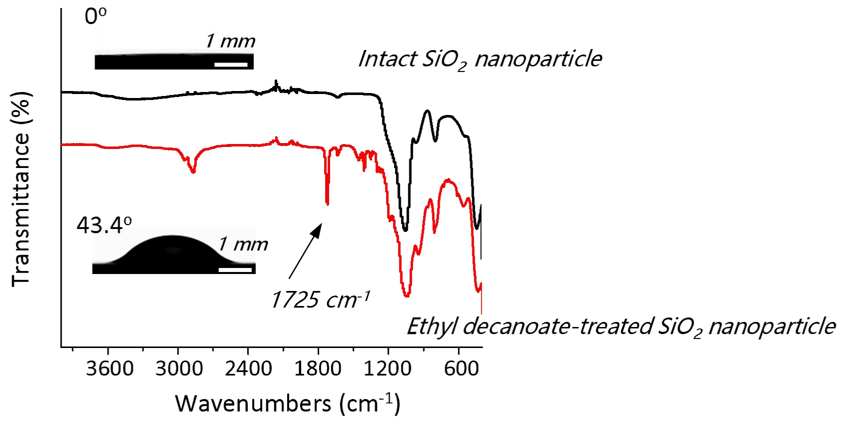

Supplementary Figure 16. FTIR spectra of intact and ethyl decanoate-treated SiO_2 nanoparticles. Inserts show the photographs of the water contact angle of the surfaces coated with intact and ethyl decanoate-treated SiO_2 nanoparticles, respectively.

As shown in **Supplementary Figure 16**, the spectrum of ethyl decanoate-treated SiO_2 nanoparticles shows a peak centered at 1725 cm^{-1}, a characteristic signal of the stretching vibration of ester bonds, in comparison to that of intact SiO_2 nanoparticles. It indicated the occurrence of the transesterification between the ethyl decanoate and the hydroxyl on the surface of SiO_2 nanoparticles during the preparation of the initiated samples (**Supplementary Figure 17**). This hypothesis was also supported by the appearance of the signals at 2950, 2874, and 1380 cm^{-1} which were the characteristic peaks of long alkyl chains[1]. The as-prepared SiO_2 nanoparticles were hydrophilic and could be completely wetted by water. After ethyl decanoate treatment, a water contact angle of 43.4° was observed on the surface of the treated SiO_2 nanoparticles.

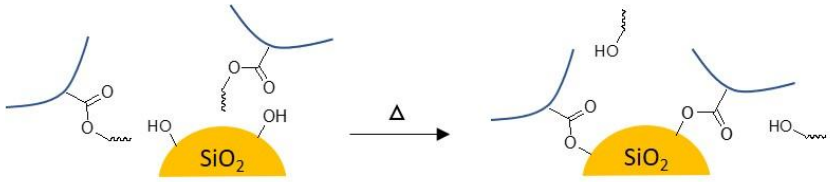

Supplementary Figure 17. Schematic diagram of transesterification occurred between hydroxyl on the surface of SiO_2 nanoparticles and polymer chains of matrices.

9. Mechanical properties and self-healing of photonic composites

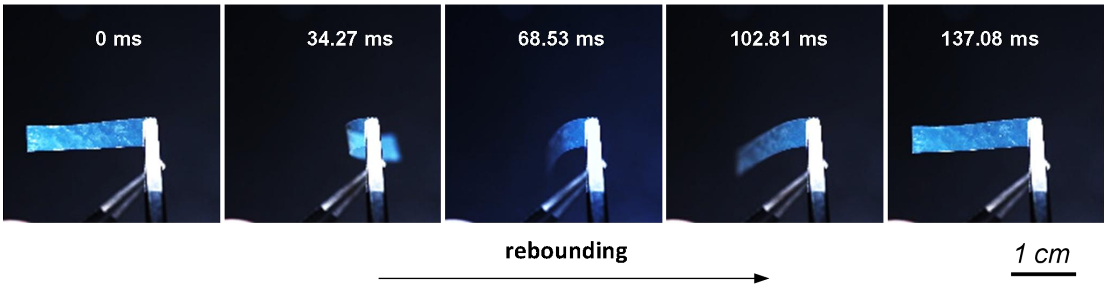

Supplementary Figure 18. Digital photos show the flexibility and elasticity of a grown sample made from the alcohol nutrient.

Growth in ethanol-containing nutrients softens and toughens the composites. We assumed that the transesterification between ethanol and polymer matrices contributed to this effect. To prove the hypothesis, we prepared SiO_2-free polymer films and allowed the films to grow in different conditions. As shown in **Supplementary Figure 19**, SiO_2-free polymer films (as-prepared PEGDA film) become softer growth in the ethanol-containing nutrient but stiffer in the ethanol-free nutrient. The softer grown sample could be stiffened by annealing to remove the ethanol moieties incorporated in the sample, showing a similar curve to the grown sample directly obtained from the ethanol-free nutrient. This stiffening result indicated the reversibility of the transesterification mechanism that was designed to achieve the reshaping of the samples.

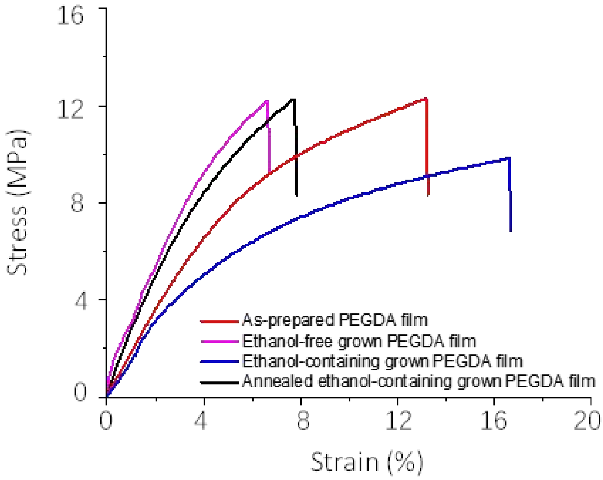

Supplementary Figure 19. Strain-stress curves of PEGDA film (as-prepared), grown PEGDA film obtained from *EG* (ethanol-free grown), grown PEGDA film obtained from ethanol-containing *EG* (ethanol-containing grown), and annealed grown PEGDA polymer obtained from ethanol-containing *EG* (annealed ethanol-containing grown).

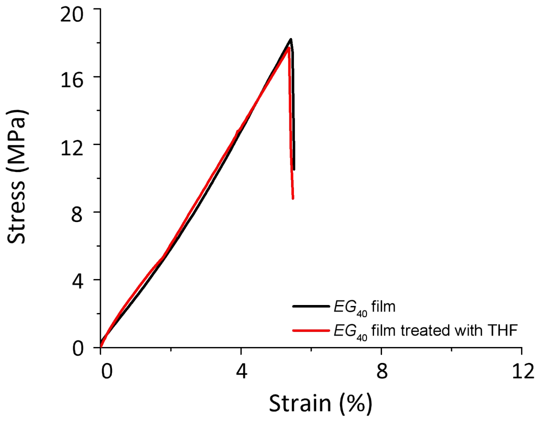

Supplementary Figure 20. Strain-stress curves of EG_{40} film and EG_{40} film immersed with tetrahydrofuran and drying.

10. Selective growth

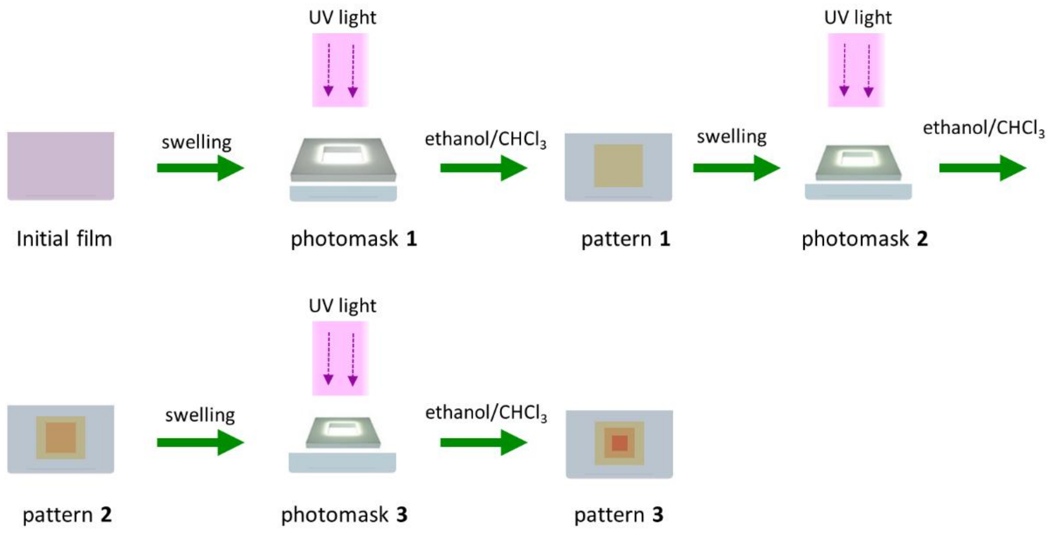

Supplementary Figure 21. A schematic image to describe the procedure of preparing patterns.

11. Fabrication of "Sichuan facebook"

Similarly, we have fabricated the “Sichuan facebook” through our growing strategy. Three photomasks with different facebook shapes (glasses deposited by chromium, see Figure S20) were employed to control the growing regions. EG_{48} with single blue color was used as the starting substrate, which was immersed in the nutrient EG for swelling (the sample became olivine). The swollen sample was placed on a glass substrate and covered by a photomask, followed by UV light irradiation (intensity: 10 mW·cm^{-2}). During irradiation, the irradiated regions became yellow. The whole sample (still covered by the photomask) was then

annealed in an oven at 70 °C for 5 h (no color change was observed during annealing). After annealing, the photomask was removed and the film was peeled off from the glass substrate. We found that the unirradiated regions were green. When we used ethanol/$CHCl_3$ solution to wash the free nutrients, the irradiated regions were still yellow but the unirradiated regions turned back to blue. This growing cycle was repeated twice again by using different photomasks, finally leading to the multicolor "Sichuan facebook" pattern.

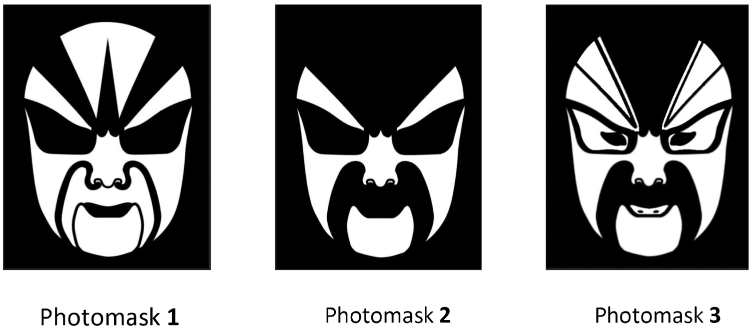

Supplementary Figure 22. Three kinds of photomasks used in the preparation of "Sichuan facebook" in sequence.

Control self-healing experiment: a sharp blade was used to make a scratch in an intact $EG_{50.3}$ film (**Supplementary Figure 23** left). Subsequently, the scratched $EG_{50.3}$ were subjected to an oven and annealed at 70°C for 12 h. The scratch cannot be self-healed (**Supplementary Figure 23** right).

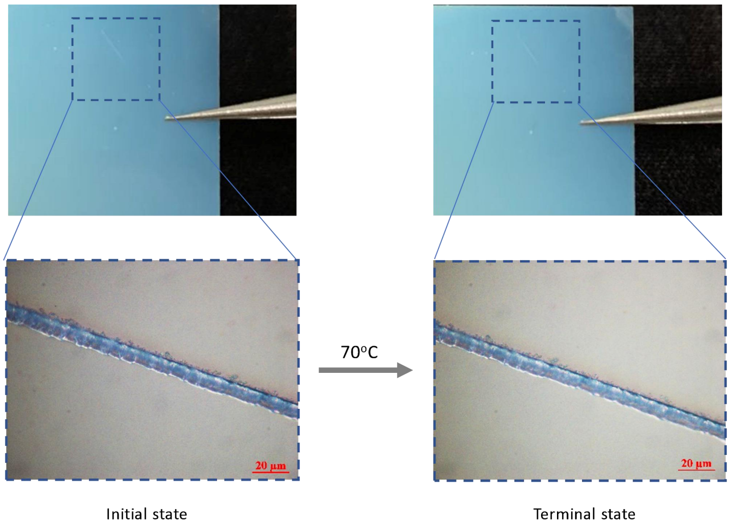

Supplementary Figure 23. Light microscope photographs of the healing process of $EG_{50.3}$ treated by heating at 70°C in an oven for 12 h. Left: intact state; Right: annealed state.

Growth-induced self-healing experiment: a similar scratch was created in an intact $EG_{50.3}$ film (**Supplementary Figure 24**). The scratched sample was immersed in EG for 5 h. For self-healing, the scratched area was exposed to UV light (intensity: 10 mW·cm^{-2}) for 2 min. Subsequently, the illuminated sample was annealed in an oven at 70°C for 2 h, followed by washing with ethanol/chloroform, to acquire the self-healed sample.

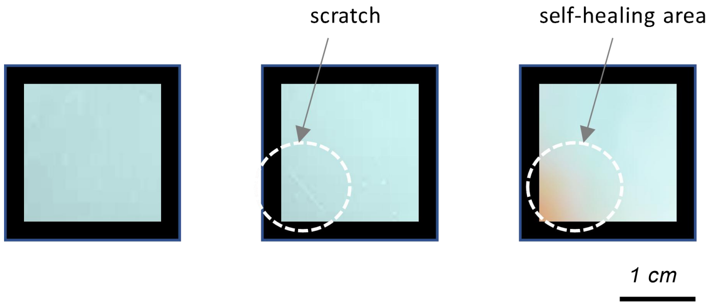

Supplementary Figure 24. Digital images of a $EG_{50.3}$ at different states: intact film (left), scratched film (middle), healed film (right).

Supplementary Reference

1 Liu, Y.-J., Navasero, N. M. & Yu, H.-Z. Structure and reactivity of mixed ω-carboxyalkyl/alkyl monolayers on silicon: ATR-FTIR spectroscopy and contact angle titration. *Langmuir* **20**, 4039-4050 (2004).
